# Hepatitis B virus DNA level Among the Seropositive Afghan Immigrants, Southern Iran

**DOI:** 10.5812/jjm.10127

**Published:** 2014-05-01

**Authors:** Mohammad Amin Behzadi, Mazyar Ziyaeyan, Sadaf Asaei

**Affiliations:** 1 Professor Alborzi Clinical Microbiology Research Center, Namazi Hospital, Shiraz University of Medical Sciences, Shiraz, IR Iran; 2 Student Research Committee, Shiraz University of Medical Sciences, Shiraz, IR Iran

**Keywords:** Hepatitis B Virus, Viral Load, Iran

## Abstract

**Background:**

Diagnosis and control programs for infectious diseases among immigrants are the most important aspects of epidemiological studies for both origin and destination countries. Data about hepatitis B virus (HBV) infection among the Afghan immigrants in Iran is limited.

**Objectives:**

To the best of HBV treatment and prevention in Afghan immigrants in Iran, the present study was conducted to determine the virus DNA level, and the frequency of respective hepatitis B risk factors among the respective seropositive patients in Fars province, southern Iran.

**Patients and Methods:**

A total of 64 HBsAg positive Afghan immigrants including 47 (73.4%) men and 17 (26.6%) women, with ages ranging between 15 and 74 years (mean ± standard deviation: 37.69 ± 15.02 years) participated in this study. From those, whole blood sample were collected and DNAs were extracted from the sera and analyzed by TaqMan real-time PCR assay with a set of primers and probe amplified core protein region of HBV genome.

**Results:**

HBV DNA was detected in a total of 51/64 (79.7 %) serum samples; 37 (72.5%) male and 14 (27.5%) female. The copy number of HBV DNA ranged from 5 × 10^2^ to 8.49 × 10^8^ copies/mL in the serum samples; median 3.8 × 10^4^ copies/mL. Demographic data and risk factors were also evaluated. The comparison of viral loads between the age groups and sex indicated no significant correlation (P > 0.05). However, the serum HBV DNA level significantly decreased in the treated patient group (P = 0.03). There was no significant difference in medicine usage between the two sexes in the study population (P > 0.05).

**Conclusions:**

Considering the results, determining the HBV DNA load and evaluation of treatment response can help to reduce the costs of diagnosis and treatment procedures in such patients, as well as, decreasing the risk of HBV transmission in immigrant Afghan population. Moreover, HBV screening strategies in country border entrances among immigrant should be performed. Moreover, free vaccination and treatment programs, and improving the level of HBV knowledge among Afghan immigrants in Iran is highly recommended.

## 1. Background

Hepatitis B virus (HBV), a hepatotrophic, double-stranded circular DNA virus, belongs to the *Hepadnaviridae* family which contains several avian and mammalian variants (1). This virus has been known as a common cause of acute and chronic hepatitis infections in humans and may lead to cirrhosis, hepatocellular carcinoma (HCC) and liver failure (2). HBV infection can be transmitted by blood products, intravenous drug abuse, unprotected sexual contact, transplantation of organs from the infected donors, during birth, and vertically from the infected mothers (3).

During the last two decades, serological, virological, biochemical, and histological diagnostic tools are used frequently to monitor and characterize the clinical state of HBV infection. One of the first serologic markers appearing after HBV infection is Hepatitis B surface antigen (HBsAg). Its persistence for more than 6 months reveals chronic HBV infection. Furthermore, serum HBV DNA level indicates the response to antiviral therapy and diagnosis of drug resistance (4, 5). HBV infection is worldwide and has been identified as a global serious public health problem. It is estimated that the disease affects 400 million people in the world with annually 600000 deaths from cirrhosis, liver failure, and HCC (6, 7). 

Chronic HBV infection is endemic in most of Eastern Europe, Asia, Africa, the Middle East, and the Pacific basin population, where it is the leading cause of cirrhosis and HCC (2, 8-10). Chronic HBV endemicity is intermediate in the Middle East where 2 to 7% are chronic carriers; however, Iran is situated in low level of endemicity map in this region with a carrier frequency of 3% (11). Epidemiological studies have indicated that chronic HBV infection is the most frequent cause of chronic hepatitis (70-80%), HCC (46%), and hepatic cirrhosis (51%) in Iran (12, 13).

Iran and other three main countries bordering Afghanistan (Pakistan, Tajikistan, and Uzbekistan) provided refuge to many Afghans during the extended period of civil war and they might have been affected by HBV infected immigrants. Thus, prevalence survey of HBV infection in such countries among both natives and incoming immigrants should be performed to optimize the strategies for controlling the disease in the region. So far, there have been limited reports on HBV infection among Afghan immigrants in Iran.

## 2. Objectives

To the best of HBV treatment and prevention in Afghan immigrants in Iran, the present study was conducted to determine the virus DNA level, and the frequency of respective hepatitis B risk factors among the respective seropositive patients in Fars province, southern Iran.

## 3. Patients and Methods

### 3.1. Namazi Hospital, Shiraz University of Medical Sciences, IR Iran

Namazi Hospital is a 1200 bed tertiary care hospital, affiliated with Shiraz University of Medical Sciences, Shiraz, Iran, which also serves as an urban general hospital in Shiraz. It serves as a referral hospital in the area with about 4 million inhabitants in south of Iran.

### 3.2. Study Population

In the HBV serological screening program, conducted by Iranian Blood Transfusion Organization among all the Afghan immigrants in Fars province, the seropositive patients were referred to Namazi Hospital for further follow up and treatment. The study population consisted of 64 Afghan immigrant individuals referred to Professor Alborzi Clinical Microbiology Research Center, Namazi Hospital, Shiraz, Iran, from November 2007 to April 2012 for HBV diagnosis. Each individual was interviewed about demographic characteristics. The 64 patients included 47 (73.4%) men and 17 (26.6%) women, with ages ranging between 15 and 74 years (mean ± standard deviation: 37.69 ± 15.02 years). The patients were divided into seven age groups: 9-19 years, 20-29 years, 30-39 years, 40-49 years, 50-59 years, 60-69 years and ≥ 70. All the patients were with suspected HBV infection and serologically positive for hepatitis B surface antigen (HBsAg).

### 3.3. Sampling and DNA Extraction

Blood samples for harvesting of serum were collected from all the patients, and left to clot at room temperature for one hour and subsequently centrifuged. The sera were separated, aliquoted, and stored at -20˚C for further examination. Viral DNA was extracted from 200μl of all serum samples by Invisorb® spin virus DNA Mini Kit (Invitek, Berlin, Germany), according to the manufacturer’s protocol. A standardized amount of internal control DNA, supplied with the real-time PCR kit, was added to the respective lysis buffer to monitor the efficiency of sample extraction. Negative and positive controls were included in the extraction process.

### 3.4. Real-Time PCR

The Real-Time quantitative PCR was performed with oligonucleotide primer pairs and probe specific for the core protein region of HBV genome by Advanced Kit (Primer Design Ltd., Millbrook Technology Campus, and Southampton, UK). Amplification was carried out in an Applied Biosystem Sequence Detector 7500 machine (Applied Biosystems, USA), using TaqMan universal real-time PCR master mix reagents (Invitrogen, Carlsbad, CA). It was programmed for a four step protocol: 2 minutes of incubation at 50˚C for AmpErase activation, 10 minutes at 95˚C for polymerase activation and for 45 cycles: 10 seconds at 95˚C for denaturation, 60 seconds at 60˚C for annealing, extension and data collection.

### 3.5. Statistical Analysis

The statistical analyses were done by SPSS for Windows (version 16, SPSS Inc., Chicago, IL, USA) and the data were considered statistically significant at a two-sided P < 0.05. The values of DNA copies/mL in the patients’ sera were first explored to find the distribution pattern in the current study population (n = 64). In doing so, one-sample Kolmogorov-Smirnov test was applied. Based on the results of this test, a significant difference (P < 0.05), indicates that the distribution is not normal. Therefore, data were analyzed by Mann‐Whitney, Kruskal‐Wallis, and Chi‐square tests. Descriptive statistics were also calculated and reported for demographic variables and risk factors including age, sex, living place, marital status, job, education, and the history of blood transfusion, surgery, addiction, prison history and HBV treatment.

## 4. Results

**Figure 1.**
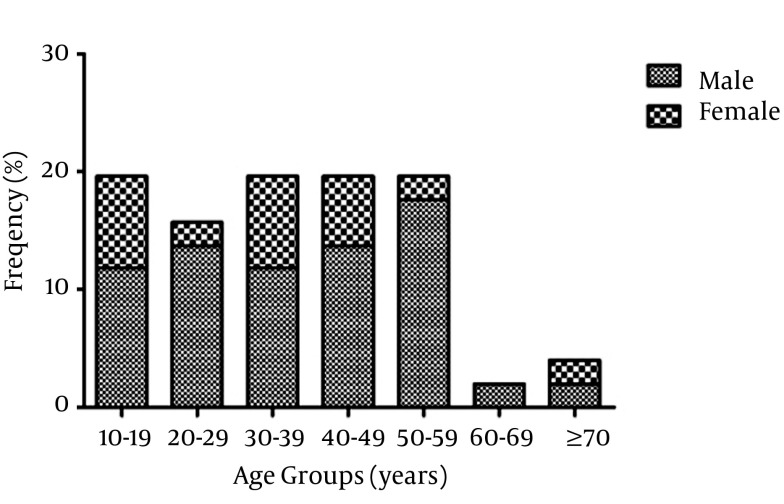
Prevalence of HBV DNA Positive Patients in Different Age Groups and Sexes Among HBV Seropositive Afghan Immigrants in Fars Province, Southern Iran

HBV DNA was detected in a total of 51/64 (79.7 %) patients’ serum samples. The copy number of HBV DNA, measured by the Real-Time PCR assay, ranged from 5 × 10^2^ to 8.49 × 10^8^ copies/mL in the samples; Median 3.8 × 10^4^ copies/mL. Of these DNA positive patients, 37 (72.5%) were males and 14 (27.5%) were females and 88.2 % were married. Other demographic data and risk factors are presented in Table 1. The results of one-sample Kolmogorov-Smirnov test indicate that the distribution of values of DNA copies/mL patients’ sera was not normal (P < 0.05). Thus, nonparametric tests were applied. The comparison of viral loads 

between the age groups and sexes indicated no significant correlation (P > 0.05) (Figure 1). However, there was a significant difference in viral load between the HBV treated group and non-treated counterpart (P = 0.03). There was no significant difference in medicine usage between the two sexes in the study population (P > 0.05).

**Table 1.** Frequency of Hepatitis B risks Factors and Demographic Variables Among HBV DNA Positive Afghan Immigrants in Fars Province, Southern Iran (n = 51) ^a^

Characteristic	Results
**Sex**	
Male	37 (72.5)
Female	14 (27.5)
**Marital status**	
Single	6 (11.8)
Married	45 (88.2)
**Living place**	
Urban	51 (100.0)
Rural	0
**Birth place**	
Iran	0
Other countries	51 (100.0)
**Education**	
≤ High school	51 (100.0)
˃ High school	0
**Injected Addiction**	
Yes	2 (3.9)
No	49 (96.1)
**Prison**	
Yes	1 (2.0)
No	50 (98.0)
**Surgery**	
Yes	11 (21.6)
No	40 (78.4)
**Blood transfusion**	
Yes	10 (19.6)
No	41 (80.4)
**Job**	
Simple worker	49 (96.1)
Other	2 (3.9)
**HBV treatment**	
Yes	15 (29.4)
No	36 (70.6)

^a^ Data are presented in No. (%).

## 5. Discussion

Although several surveys have been performed on HBV infection in native individuals in Iran (14, 15), there is limited information about the disease among Afghan immigrants (16). To our knowledge, this is the first report on HBV DNA level among Afghan immigrants in Iran.

In this study, HBV DNA was detected in 79.9 % of seropositive Afghan patients. No mode of HBV transmission among them was detected; however, the risk factors were evaluated. It was shown that one of the main transmission routes of the disease in North America and Europe is through blood and exposure to contaminated needles (17). Also, the results of our study showed that 21.6 % and 19.6% of the patients had the history of surgical procedures and blood transfusion, respectively. Due to very limited supply of disposable needles and syringes inside prison, handmade injection devices and sharing needles are used frequently by addict prisoner in Iran. This could be the common route of spreading the infection among the prisoners. A previous study in Afghanistan indicated that the prevalence of HBsAg among adult injection drug users in Kabul was 6.5% (18). In the present study, 3.9 % of the patients were addicted to injection drugs and 2% were prisoner in Iran.

Bulks of related literature maintain that individual education seems to play an important role in prevention, distribution and treatment of HBV infection (19-21). As expected, the entire patients in the present study had less than high school education and the majority of them (96.1 %) were simple workers. Similar level of knowledge of HBV has been also found among local Asian- Pacific individuals (22). Previous studies showed that contracting HBV during birth and early childhood (˂ 5 years), is one of the common routes of infection transmission among patients in Asia and Africa where the incidence is higher than 8% (8, 23, 24). It was also manifested that the risk of chronic infection in future for HBV infected neonates and children (younger than 1 year) is 90% (25). 

The ratio of HBs Ag positivity might represent chronically HBV carriers (26). All the patients in this investigation were HBs Ag positive and they might have been with chronic infection. The results of our study indicated that all the DNA positive patients were born outside Iran and it may be concluded that they were already infected at the time of immigration. It was shown that the seropositivity rate has increased with age; however, HBs Ag positivity has not increased the same (26). Similarly, in this study, the viral loads did not increase significantly with age (P > 0.05). Moreover, no association was found between HBV viral load and the gender of patients (P > 0.05).

The results of our study revealed that 72.5% of HBV DNA positive patients were male, which is in agreement with an earlier report in Turkey (26). It is confirmed that the infection can be transmitted to adults during unprotected sex with infected partners. Multi partnership is unusual among females in Islamic countries. In addition, other risk factors such as intravenous illicit drug usage are more common in males than females. These may be the explanation for the high prevalence of disease among male Afghan immigrants in Iran. Detection of HBV DNA in serum can define the state of the infection in respective patients. According to the HBV infection phase, several protocols are recommended for the treatment of the disease. However, due to the high costs of the medications including HBIG, the immigrant population in this study did not use them. 

The only medicine they were treated with was lamivudine. According to the results of present investigation, the serum HBV DNA level significantly decreased in the treated patient group (P = 0.03). The majority of the HBV DNA negative patients (12/13) were treated with lamivudine monotherapy. It seems that current treatment is effective to control the disease. However, the majority of patients was not referred to physicians and not treated (70.6%). This may be related to the lack of knowledge about the disease and low financial status of the patients’ families. No significant difference was found in medicine usage between the sexes in the study population (P > 0.05). The results of this study demonstrated that all the HBV DNA positive Afghan immigrants were living in urban areas with crowded population. It is quite alarming that it could be a predisposing factor in spreading of the infection in the cities.

A limitation of this study was the lack of information about the history of infection in patients’ families and their sexual patterns. Many Afghan immigrants were afraid of disclosing correct information, because of risk of being arrested, or deported to their country of origin. In conclusion, determining the HBV viral load in such group will be helpful for;

1. Appropriate treatment including types of medication and duration;
2. Evaluation of the treatment response and finding the drug resistance pattern in such patients;
3. Prevention and controlling of the infection in immigrant Afghan population.

However, additional investigations are needed to determine the prevalence of HBV infection among them in other parts of Iran and evaluate the potential risk factors in the distribution of the infection in this population. Although HBV screening strategies are currently pursued in many parts of Iran, they are still inadequate especially in border entrances. Moreover, free nationwide vaccination programs and treatment for this population are highly recommended. Improving the education about HBV among Afghan immigrants in Iran, could be also helpful to effective control and prevention of the disease in future.
